# 2,2′-({4-[(4-Nitro­phen­yl)diazen­yl]phen­yl}imino)­diethanol

**DOI:** 10.1107/S1600536812049239

**Published:** 2012-12-05

**Authors:** Tanwawan Duangthongyou, Potjanart Suwanruji, Jantip Suesat, Supakit Achiwawanich

**Affiliations:** a Department of Chemistry, Faculty of Science, Kasetsart University, Bangkok 10903, Thailand; b Department of Textile Science, Faculty of Agro-Industry, Kasetsart University, Bangkok 10900, Thailand

## Abstract

In the title compound, C_16_H_18_N_4_O_4_, the mol­ecule assumes an *E* conformation with respect to the N=N double bond. The aromatic rings are not coplanar, with a dihedral angle of 7.51 (8)°. The nitro group is tilted by 4.71 (11)° relative to the attached benzene ring. In the crystal, mol­ecules are connected through O—H⋯O hydrogen bonds forming a double-stranded chain parallel to the *b* axis.

## Related literature

For the properties of azo disperse dyes, see: Suesat *et al.* (2011 ▶). For the structure of related compounds, see: Zhang *et al.* (1998 ▶); Adams *et al.* (2004 ▶).
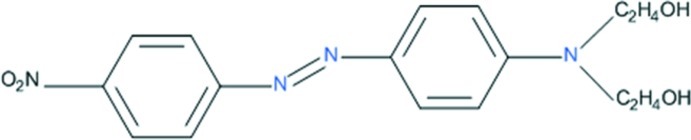


## Experimental

### 

#### Crystal data

C_16_H_18_N_4_O_4_
*M* *_r_* = 330.34
Monoclinic, 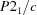

*a* = 19.000 (3) Å
*b* = 7.3502 (16) Å
*c* = 11.0825 (16) Å
β = 92.060 (8)°
*V* = 1546.7 (5) Å^3^
*Z* = 4
Mo *K*α radiation
μ = 0.10 mm^−1^
*T* = 296 K
0.24 × 0.16 × 0.04 mm

#### Data collection

Bruker APEXII CCD diffractometer
7008 measured reflections
2671 independent reflections
1642 reflections with *I* > 2σ(*I*)
*R*_int_ = 0.032

#### Refinement

*R*[*F* ^2^ > 2σ(*F* ^2^)] = 0.044
*wR*(*F* ^2^) = 0.152
*S* = 0.93
2671 reflections
219 parameters
H-atom parameters constrained
Δρ_max_ = 0.17 e Å^−3^
Δρ_min_ = −0.19 e Å^−3^

### 

Data collection: *APEX2* (Bruker, 2011 ▶); cell refinement: *SAINT* (Bruker, 2011 ▶); data reduction: *SAINT*; program(s) used to solve structure: *SHELXS97* (Sheldrick, 2008 ▶); program(s) used to refine structure: *SHELXL97* (Sheldrick, 2008 ▶); molecular graphics: *SHELXTL* (Sheldrick, 2008 ▶); software used to prepare material for publication: *SHELXTL*.

## Supplementary Material

Crystal structure: contains datablock(s) I, global. DOI: 10.1107/S1600536812049239/rz5028sup1.cif

Structure factors: contains datablock(s) I. DOI: 10.1107/S1600536812049239/rz5028Isup2.hkl

Supplementary material file. DOI: 10.1107/S1600536812049239/rz5028Isup3.cml

Additional supplementary materials:  crystallographic information; 3D view; checkCIF report

## Figures and Tables

**Table 1:** Hydrogen-bond geometry (Å, °)

*D*—H⋯*A*	*D*—H	H⋯*A*	*D*⋯*A*	*D*—H⋯*A*
O1—H1⋯O4^i^	0.82	1.90	2.700 (3)	164
O4—H4⋯O1^ii^	0.82	1.90	2.718 (3)	172

Symmetry codes: (i) 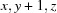; (ii) 
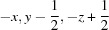
.

**Fig. 1.**
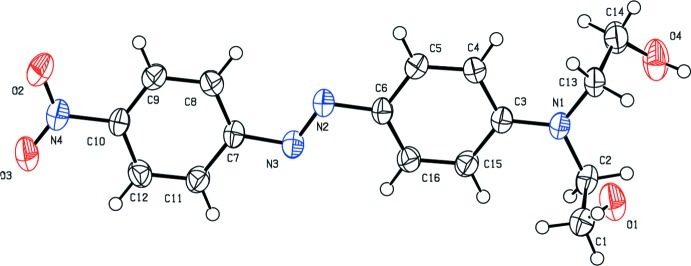
The molecular structure of the title compound showing displacement ellipsoids drawn at the 50% probability level.

**Fig. 2.**
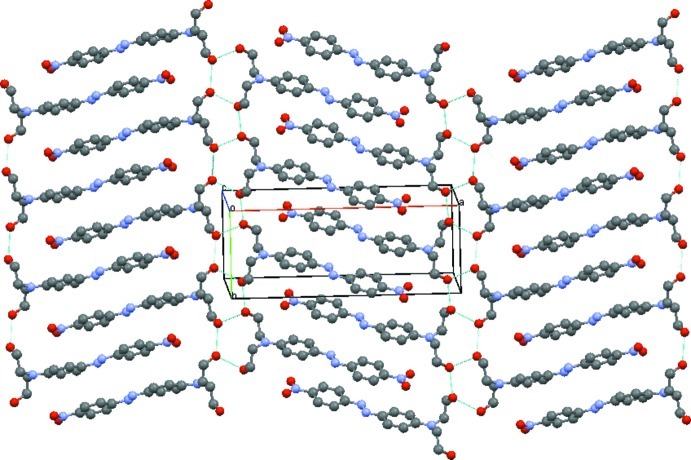
The chain structure of the title compound. Hydrogen bonds are shown as dotted lines.

## supplementary crystallographic information

### Comment

A series of azo disperse dyes was recently synthesized by our group in order to study the influence of substituents on the chromatic properties of the dyes (Suesat *et al.*, 2011). We report herein the crystal structure of one of these dyes.

The molecule of the title compound (Fig. 1) displays an *E* configuration about the N═N double bond and is not planar, the dihedral angle between the aromatic ring being 7.51 (8)°. This value may be compared with those observed in the related compounds 4'-(dimethylamino)-2-nitroazobenzene (5.3 (2)°; Zhang *et al.*, 1998) and 4'-(dimethylamino)-4-nitroazobenzene (2.1 (4)°; Adams *et al.*, 2004). The nitro group is tilted by 4.71 (11)° with respect to the attached C7–C11 benzene ring. In the crystal structure, molecules are linked by O—H···O hydrogen bonds (Table 1) to form double-stranded chain parallel to the *b* axis (Fig. 2).

### Experimental

The azo disperse dye was prepared by dissolving 4-nitroaniline (0.01 mol) in 50 ml of an acetic acid/propionic acid (43:7 *v*/*v*) mixture. The solution was stirred and the temperature was kept in the range of 0–5°C. Diazotization took place when nitrosyl sulfuric acid (HNO_5_S) was added to the solution and stirred at 0–5°C for 30–60 minutes. The coupling component *N*-bis-β-hydroxyethyl aniline (0.01 mol) was then dissolved in 40 ml acetone, distilled water was added to make the total volume of 200 ml and sulfamic acid (0.5 g) was added. The coupling reaction was performed by slow addition of the diazonium salt solution to the coupling solution at 0–5°C. The reaction continued for 2 h with stirring and was monitored using TLC. On completion of the coupling reaction, the dye precipitate was collected by filtration and dried at room temperature. The dye was purified by recrystallization in n-propanol. Single crystals suitable for X-ray analysis were obtained by slow evaporation of a purified dye solution in n-propanol.

### Refinement

All H atoms of the compound were placed in the calculated positions with C—H = 0.93 and 0.97 Å, O—H = 0.82 Å and included in the cycles of refinement in a rigid model, with *U*_iso_(H) = 1.2 *U*_eq_(C) and 1.5 *U*_eq_(O).

### Crystal data

**Table:**

C_16_H_18_N_4_O_4_	*F*(000) = 696
*M**_r_* = 330.34	*D*_x_ = 1.419 Mg m^−^^3^
Monoclinic, *P*2_1_/*c*	Mo *K*α radiation, λ = 0.71073 Å
Hall symbol: -P 2ybc	Cell parameters from 1201 reflections
*a* = 19.000 (3) Å	θ = 3.0–21.9°
*b* = 7.3502 (16) Å	µ = 0.10 mm^−^^1^
*c* = 11.0825 (16) Å	*T* = 296 K
β = 92.060 (8)°	Plate, purple
*V* = 1546.7 (5) Å^3^	0.24 × 0.16 × 0.04 mm
*Z* = 4	

### Data collection

**Table:**

Bruker APEXII CCD diffractometer	1642 reflections with *I* > 2σ(*I*)
Radiation source: fine-focus sealed tube	*R*_int_ = 0.032
Graphite monochromator	θ_max_ = 25.1°, θ_min_ = 1.1°
φ and ω scans	*h* = −22→22
7008 measured reflections	*k* = −8→7
2671 independent reflections	*l* = −13→12

### Refinement

**Table:**

Refinement on *F*^2^	Primary atom site location: structure-invariant direct methods
Least-squares matrix: full	Secondary atom site location: difference Fourier map
*R*[*F*^2^ > 2σ(*F*^2^)] = 0.044	Hydrogen site location: inferred from neighbouring sites
*wR*(*F*^2^) = 0.152	H-atom parameters constrained
*S* = 0.93	*w* = 1/[σ^2^(*F*_o_^2^) + (0.096*P*)^2^] where *P* = (*F*_o_^2^ + 2*F*_c_^2^)/3
2671 reflections	(Δ/σ)_max_ < 0.001
219 parameters	Δρ_max_ = 0.17 e Å^−^^3^
0 restraints	Δρ_min_ = −0.19 e Å^−^^3^

### Special details

**Table:**

Geometry. All e.s.d.'s (except the e.s.d. in the dihedral angle between two l.s. planes) are estimated using the full covariance matrix. The cell e.s.d.'s are taken into account individually in the estimation of e.s.d.'s in distances, angles and torsion angles; correlations between e.s.d.'s in cell parameters are only used when they are defined by crystal symmetry. An approximate (isotropic) treatment of cell e.s.d.'s is used for estimating e.s.d.'s involving l.s. planes.
Refinement. Refinement of *F*^2^ against ALL reflections. The weighted *R*-factor *wR* and goodness of fit *S* are based on *F*^2^, conventional *R*-factors *R* are based on *F*, with *F* set to zero for negative *F*^2^. The threshold expression of *F*^2^ > σ(*F*^2^) is used only for calculating *R*-factors(gt) *etc*. and is not relevant to the choice of reflections for refinement. *R*-factors based on *F*^2^ are statistically about twice as large as those based on *F*, and *R*- factors based on ALL data will be even larger.

### Fractional atomic coordinates and isotropic or equivalent isotropic displacement parameters (Å^2^)

**Table:**

	*x*	*y*	*z*	*U*_iso_*/*U*_eq_	
O1	0.05195 (11)	0.8516 (3)	0.17672 (18)	0.0594 (6)	
H1	0.0644	0.9565	0.1905	0.089*	
O2	0.76929 (9)	0.9038 (3)	−0.02037 (17)	0.0597 (6)	
O3	0.74369 (9)	0.9967 (3)	−0.20053 (17)	0.0586 (6)	
O4	0.06568 (10)	0.2095 (3)	0.22729 (19)	0.0633 (6)	
H4	0.0285	0.2525	0.2496	0.095*	
N1	0.14613 (9)	0.5386 (3)	0.14546 (17)	0.0354 (5)	
N2	0.42757 (9)	0.6797 (3)	0.04905 (17)	0.0365 (5)	
N3	0.44265 (10)	0.7473 (3)	−0.05171 (17)	0.0368 (5)	
N4	0.72717 (11)	0.9329 (3)	−0.1042 (2)	0.0411 (6)	
C1	0.07605 (14)	0.7966 (4)	0.0624 (2)	0.0497 (8)	
H1A	0.1193	0.8604	0.0453	0.060*	
H1B	0.0410	0.8267	−0.0003	0.060*	
C2	0.08899 (12)	0.5953 (4)	0.0634 (2)	0.0408 (7)	
H2A	0.0462	0.5340	0.0858	0.049*	
H2B	0.0995	0.5566	−0.0176	0.049*	
C3	0.21506 (12)	0.5627 (3)	0.1166 (2)	0.0317 (6)	
C4	0.27042 (12)	0.5308 (3)	0.2010 (2)	0.0367 (6)	
H4A	0.2606	0.4825	0.2761	0.044*	
C5	0.33886 (12)	0.5695 (4)	0.1749 (2)	0.0379 (6)	
H5	0.3743	0.5498	0.2335	0.046*	
C6	0.35640 (11)	0.6375 (3)	0.06305 (19)	0.0320 (6)	
C7	0.51515 (12)	0.7925 (3)	−0.0583 (2)	0.0332 (6)	
C8	0.56560 (12)	0.7559 (4)	0.0318 (2)	0.0406 (7)	
H8	0.5525	0.6992	0.1026	0.049*	
C9	0.63476 (13)	0.8029 (4)	0.0174 (2)	0.0403 (7)	
H9	0.6687	0.7776	0.0776	0.048*	
C10	0.65295 (11)	0.8877 (3)	−0.0873 (2)	0.0328 (6)	
C11	0.53504 (12)	0.8809 (4)	−0.1621 (2)	0.0398 (7)	
H11	0.5013	0.9080	−0.2223	0.048*	
C12	0.60435 (13)	0.9292 (4)	−0.1773 (2)	0.0399 (7)	
H12	0.6177	0.9885	−0.2470	0.048*	
C13	0.12743 (13)	0.4915 (4)	0.2676 (2)	0.0400 (6)	
H13A	0.0822	0.5462	0.2834	0.048*	
H13B	0.1620	0.5446	0.3236	0.048*	
C14	0.12301 (14)	0.2927 (4)	0.2926 (3)	0.0536 (8)	
H14A	0.1666	0.2347	0.2708	0.064*	
H14B	0.1174	0.2741	0.3784	0.064*	
C15	0.23411 (12)	0.6236 (4)	0.0017 (2)	0.0371 (6)	
H15	0.1991	0.6398	−0.0582	0.045*	
C16	0.30250 (12)	0.6596 (3)	−0.0241 (2)	0.0353 (6)	
H16	0.3132	0.6994	−0.1010	0.042*	

### Atomic displacement parameters (Å^2^)

**Table:**

	*U*^11^	*U*^22^	*U*^33^	*U*^12^	*U*^13^	*U*^23^
O1	0.0536 (12)	0.0370 (13)	0.0894 (15)	−0.0012 (10)	0.0307 (11)	−0.0022 (10)
O2	0.0321 (11)	0.0772 (16)	0.0690 (13)	−0.0058 (10)	−0.0091 (10)	0.0015 (11)
O3	0.0404 (11)	0.0767 (16)	0.0600 (13)	−0.0067 (10)	0.0176 (9)	0.0071 (11)
O4	0.0494 (13)	0.0405 (13)	0.1017 (16)	−0.0089 (10)	0.0257 (12)	−0.0144 (11)
N1	0.0246 (10)	0.0402 (14)	0.0417 (11)	−0.0018 (9)	0.0043 (9)	−0.0004 (9)
N2	0.0280 (12)	0.0409 (14)	0.0410 (12)	−0.0022 (10)	0.0065 (9)	−0.0014 (10)
N3	0.0286 (12)	0.0438 (14)	0.0380 (12)	−0.0035 (10)	0.0042 (9)	−0.0029 (10)
N4	0.0296 (12)	0.0413 (14)	0.0527 (13)	−0.0019 (10)	0.0063 (11)	−0.0080 (11)
C1	0.0353 (15)	0.056 (2)	0.0587 (17)	0.0061 (14)	0.0085 (13)	0.0114 (14)
C2	0.0262 (13)	0.0494 (18)	0.0468 (15)	−0.0054 (12)	0.0009 (11)	−0.0059 (13)
C3	0.0281 (13)	0.0275 (14)	0.0398 (13)	−0.0012 (11)	0.0041 (10)	−0.0048 (11)
C4	0.0329 (14)	0.0404 (17)	0.0370 (13)	−0.0025 (12)	0.0052 (11)	0.0026 (11)
C5	0.0284 (13)	0.0441 (17)	0.0410 (14)	−0.0009 (12)	−0.0019 (10)	0.0024 (12)
C6	0.0276 (13)	0.0323 (15)	0.0367 (13)	−0.0011 (11)	0.0066 (10)	−0.0044 (11)
C7	0.0263 (13)	0.0388 (16)	0.0346 (13)	−0.0017 (11)	0.0048 (10)	−0.0064 (11)
C8	0.0349 (15)	0.0511 (19)	0.0360 (13)	−0.0032 (13)	0.0040 (11)	0.0072 (12)
C9	0.0316 (14)	0.0484 (18)	0.0406 (14)	−0.0008 (12)	−0.0034 (11)	0.0018 (12)
C10	0.0252 (12)	0.0348 (15)	0.0386 (14)	−0.0001 (11)	0.0053 (10)	−0.0076 (11)
C11	0.0306 (14)	0.0571 (19)	0.0318 (13)	0.0000 (13)	0.0004 (10)	−0.0003 (12)
C12	0.0379 (14)	0.0500 (18)	0.0323 (13)	−0.0045 (13)	0.0074 (11)	0.0006 (12)
C13	0.0287 (13)	0.0439 (17)	0.0479 (15)	−0.0041 (12)	0.0095 (11)	−0.0005 (12)
C14	0.0384 (16)	0.0450 (19)	0.0784 (19)	−0.0011 (14)	0.0154 (14)	0.0106 (15)
C15	0.0303 (13)	0.0440 (17)	0.0368 (13)	−0.0008 (12)	−0.0006 (10)	−0.0012 (11)
C16	0.0332 (14)	0.0400 (16)	0.0331 (12)	0.0016 (12)	0.0058 (11)	−0.0006 (11)

### Geometric parameters (Å, º)

**Table:**

O1—C1	1.421 (3)	C5—C6	1.389 (3)
O1—H1	0.8200	C5—H5	0.9300
O2—N4	1.224 (3)	C6—C16	1.392 (3)
O3—N4	1.217 (2)	C7—C11	1.386 (3)
O4—C14	1.424 (3)	C7—C8	1.386 (3)
O4—H4	0.8200	C8—C9	1.373 (3)
N1—C3	1.371 (3)	C8—H8	0.9300
N1—C2	1.452 (3)	C9—C10	1.373 (3)
N1—C13	1.454 (3)	C9—H9	0.9300
N2—N3	1.265 (3)	C10—C12	1.369 (3)
N2—C6	1.401 (3)	C11—C12	1.380 (3)
N3—C7	1.422 (3)	C11—H11	0.9300
N4—C10	1.467 (3)	C12—H12	0.9300
C1—C2	1.500 (4)	C13—C14	1.490 (4)
C1—H1A	0.9700	C13—H13A	0.9700
C1—H1B	0.9700	C13—H13B	0.9700
C2—H2A	0.9700	C14—H14A	0.9700
C2—H2B	0.9700	C14—H14B	0.9700
C3—C4	1.403 (3)	C15—C16	1.367 (3)
C3—C15	1.409 (3)	C15—H15	0.9300
C4—C5	1.372 (3)	C16—H16	0.9300
C4—H4A	0.9300		
			
C1—O1—H1	109.5	C11—C7—N3	116.5 (2)
C14—O4—H4	109.5	C8—C7—N3	124.3 (2)
C3—N1—C2	121.05 (19)	C9—C8—C7	120.5 (2)
C3—N1—C13	121.1 (2)	C9—C8—H8	119.8
C2—N1—C13	116.69 (18)	C7—C8—H8	119.8
N3—N2—C6	115.78 (19)	C10—C9—C8	118.9 (2)
N2—N3—C7	112.83 (19)	C10—C9—H9	120.5
O3—N4—O2	123.5 (2)	C8—C9—H9	120.5
O3—N4—C10	118.6 (2)	C12—C10—C9	122.2 (2)
O2—N4—C10	118.0 (2)	C12—C10—N4	118.9 (2)
O1—C1—C2	109.4 (2)	C9—C10—N4	118.9 (2)
O1—C1—H1A	109.8	C12—C11—C7	120.8 (2)
C2—C1—H1A	109.8	C12—C11—H11	119.6
O1—C1—H1B	109.8	C7—C11—H11	119.6
C2—C1—H1B	109.8	C10—C12—C11	118.4 (2)
H1A—C1—H1B	108.2	C10—C12—H12	120.8
N1—C2—C1	114.0 (2)	C11—C12—H12	120.8
N1—C2—H2A	108.8	N1—C13—C14	115.1 (2)
C1—C2—H2A	108.8	N1—C13—H13A	108.5
N1—C2—H2B	108.8	C14—C13—H13A	108.5
C1—C2—H2B	108.8	N1—C13—H13B	108.5
H2A—C2—H2B	107.7	C14—C13—H13B	108.5
N1—C3—C4	121.5 (2)	H13A—C13—H13B	107.5
N1—C3—C15	122.1 (2)	O4—C14—C13	111.9 (2)
C4—C3—C15	116.4 (2)	O4—C14—H14A	109.2
C5—C4—C3	121.2 (2)	C13—C14—H14A	109.2
C5—C4—H4A	119.4	O4—C14—H14B	109.2
C3—C4—H4A	119.4	C13—C14—H14B	109.2
C4—C5—C6	121.5 (2)	H14A—C14—H14B	107.9
C4—C5—H5	119.2	C16—C15—C3	121.9 (2)
C6—C5—H5	119.2	C16—C15—H15	119.1
C5—C6—C16	117.9 (2)	C3—C15—H15	119.1
C5—C6—N2	116.2 (2)	C15—C16—C6	120.9 (2)
C16—C6—N2	125.9 (2)	C15—C16—H16	119.6
C11—C7—C8	119.2 (2)	C6—C16—H16	119.6
			
C6—N2—N3—C7	178.01 (19)	C8—C9—C10—C12	0.8 (4)
C3—N1—C2—C1	−76.6 (3)	C8—C9—C10—N4	−178.2 (2)
C13—N1—C2—C1	91.0 (3)	O3—N4—C10—C12	−4.0 (3)
O1—C1—C2—N1	−65.8 (3)	O2—N4—C10—C12	176.2 (2)
C2—N1—C3—C4	171.5 (2)	O3—N4—C10—C9	175.0 (2)
C13—N1—C3—C4	4.5 (3)	O2—N4—C10—C9	−4.8 (3)
C2—N1—C3—C15	−7.4 (3)	C8—C7—C11—C12	1.3 (4)
C13—N1—C3—C15	−174.4 (2)	N3—C7—C11—C12	−179.5 (2)
N1—C3—C4—C5	−174.5 (2)	C9—C10—C12—C11	−1.1 (4)
C15—C3—C4—C5	4.4 (3)	N4—C10—C12—C11	178.0 (2)
C3—C4—C5—C6	−1.6 (4)	C7—C11—C12—C10	0.0 (4)
C4—C5—C6—C16	−2.2 (4)	C3—N1—C13—C14	−90.9 (3)
C4—C5—C6—N2	177.1 (2)	C2—N1—C13—C14	101.5 (3)
N3—N2—C6—C5	−177.7 (2)	N1—C13—C14—O4	−67.5 (3)
N3—N2—C6—C16	1.5 (4)	N1—C3—C15—C16	175.4 (2)
N2—N3—C7—C11	−174.9 (2)	C4—C3—C15—C16	−3.6 (4)
N2—N3—C7—C8	4.2 (3)	C3—C15—C16—C6	−0.1 (4)
C11—C7—C8—C9	−1.5 (4)	C5—C6—C16—C15	3.0 (4)
N3—C7—C8—C9	179.4 (2)	N2—C6—C16—C15	−176.2 (2)
C7—C8—C9—C10	0.5 (4)		

### Hydrogen-bond geometry (Å, º)

**Table:**

*D*—H···*A*	*D*—H	H···*A*	*D*···*A*	*D*—H···*A*
O1—H1···O4^i^	0.82	1.90	2.700 (3)	164
O4—H4···O1^ii^	0.82	1.90	2.718 (3)	172

Symmetry codes: (i) *x*, *y*+1, *z*; (ii) −*x*, *y*−1/2, −*z*+1/2.

## References

[bb1] Adams, H., Allen, R. W. K., Chin, J., O’Sullivan, B., Styring, P. & Sutton, L. R. (2004). *Acta Cryst.* E**60**, o289–o290.

[bb2] Bruker (2011). *APEX2* and *SAINT* Bruker AXS Inc., Madison, Wisconsin, USA.

[bb3] Sheldrick, G. M. (2008). *Acta Cryst.* A**64**, 112–122. 10.1107/S0108767307043930 18156677

[bb4] Suesat, J., Mungmeechai, T., Suwanruji, P., Parasuk, W., Taylor, J. A. & Phillips, D. A. S. (2011). *Color Technol.* **127**, 217–222.

[bb5] Zhang, D.-C., Ge, L.-Q., Fei, Z.-H., Zhang, Y.-Q. & Yu, K.-B. (1998). *Acta Cryst.* C**54**, 1909–1911.

